# Characterization of Bacterial Communities on Trout Skin and Eggs in Relation to *Saprolegnia parasitica* Infection Status

**DOI:** 10.3390/microorganisms12081733

**Published:** 2024-08-22

**Authors:** Dora Pavić, Sunčana Geček, Anđela Miljanović, Dorotea Grbin, Ana Bielen

**Affiliations:** 1 Faculty of Food Technology and Biotechnology, University of Zagreb, Pierottijeva 6, 10000 Zagreb, Croatia; dora.pavic@pbf.unizg.hr (D.P.); andela.miljanovic@gmail.com (A.M.); dorotea.polo@gmail.com (D.G.); 2 Ruđer Bošković Institute, Bijenička cesta 54, 10000 Zagreb, Croatia; suncana.gecek@irb.hr; 3 Croatian Veterinary Institute, Savska cesta 143, 10000 Zagreb, Croatia

**Keywords:** saprolegniosis, aquaculture, host-associated microbiome, trout

## Abstract

We have investigated the changes in the microbial communities on the surface of trout eggs and the skin of adult trout in relation to the presence of *Saprolegnia parasitica*. This pathogen causes saprolegniosis, a disease responsible for significant losses in salmonid farms and hatcheries. It is known from other disease systems that the host-associated microbiome plays a crucial role in the defence against pathogens, but if the pathogen predominates, this can lead to dysbiosis. However, analyses of the effects of *S. parasitica* on the diversity, composition, and function of microbial communities on fish skin and eggs are scarce. Thus, we have collected skin swabs from injured and healthy trout (*N* = 12), which differed in *S. parasitica* load, from three different fish farms in Croatia (Kostanjevac, Radovan, and Solin), while trout egg samples (*N* = 12) were infected with *S. parasitica* in the laboratory. Illumina sequencing of the V4 region of the *16S* rRNA marker gene showed that infection with *S. parasitica* reduced the microbial diversity on the surface of the eggs, as evidenced by decreased Pielou’s evenness and Shannon’s indices. We further determined whether the bacterial genera with a relative abundance of >5.0% in the egg/skin samples were present at significantly different abundances in relation to the presence of *S. parasitica*. The results have shown that some genera, such as *Pseudomonas* and *Flavobacterium*, decreased significantly in the presence of the pathogen on the egg surface. On the other hand, some bacterial taxa, such as *Acinetobacter* and *Janthinobacterium*, as well as *Aeromonas*, were more abundant on the diseased eggs and the injured trout skin, respectively. Finally, beta diversity analyses (weighted UniFrac, unweighted UniFrac, Bray–Curtis) have shown that the sampling location (i.e., fish farm), along with *S. parasitica* infection status, also has a significant influence on the microbial communities’ composition on the trout skin and eggs, demonstrating the strong influence of the environment on the shaping of the host surface microbiome. Overall, we have shown that the presence of *S. parasitica* was associated with changes in the diversity and structure of the trout skin/egg microbiome. The results obtained could support the development of new strategies for the management of saprolegniosis in aquaculture.

## 1. Introduction

The host-associated microbiome, including the microbial communities that reside in the biofilms on the surface of fish skin and eggs, plays a crucial role in defending animals against disease-causing pathogens [1,2,3]. When the skin-penetrating pathogenic microbes attempt to invade the host, they encounter the resident microbial communities and enter various ecological interactions with them, including competition for resources and direct inhibition by the production of diverse antimicrobial compounds [3,4]. On the other hand, if the pathogen prevails, it can outcompete and displace beneficial microbes, leading to dysbiosis, i.e., changes in the diversity, composition, and function of host-associated microbiome [5]. Understanding how microbiomes change in relation to pathogen presence can offer valuable insights into disease ecology. Here, we have chosen to study this question by focusing on the skin-penetrating pathogens of trout.

A number of microbes and parasites cause egg infections and skin diseases of adult trout. Such skin-infecting pathogens are of great importance in trout aquaculture, as they entail considerable risks, such as impaired health, reduced growth rates, increased susceptibility to secondary infections, and high mortality rates, all of which are associated with high economic losses. Skin diseases in trout aquaculture can be caused by various bacterial species, such as Columnaris disease, caused by *Flavobacterium columnare* [6]; Aeromonas septicemia, caused by *Aeromonas hydrophila* [7]; and furunculosis, caused by *A. salmonicida* [8]. Parasites, such as the ciliate *Ichthyophthirius multifiliis* [9] and the monogenean *Gyrodactylus* spp. [10], are also known to cause skin damage. However, one of the most important skin-penetrating diseases in trout aquaculture and the disease that causes by far the highest egg mortality and a reduction in hatching rates is saprolegniosis [11,12,13]. This disease, caused by water moulds (Oomycota) from the genus *Saprolegnia*, usually involves infection of the skin, and typically starts at the locations of lesions/injuries [14]. In the case of egg infection, the mycelium first invades dead or unfertilized eggs and then quickly spreads to the neighbouring healthy eggs, often resulting in the loss of the entire egg batches in hatcheries [15]. Saprolegniosis is typically considered as a secondary infection, since it mostly affects already immunocompromised individuals, i.e., those with injuries, prior infections by other pathogens, or under stress [13,16]. The environmental factor that is most frequently linked to *Saprolegnia* outbreaks is water temperature, which is in line with the fact that the immune defence is temperature-dependent in its stenotherm hosts [17,18,19,20]. Among *Saprolegnia* species, *S. parasitica* is considered to be the most virulent, sometimes being responsible for primary infections, and is the one that causes most of the disease outbreaks in aquaculture facilities [21].

Previous studies have shown that oomycete infections can induce significant changes in the host-associated microbiome. For instance, the infection of the obligate oomycete pathogen *Albugo* was shown to significantly alter the structure of the microbial communities of *Arabidopsis thaliana* leaves. The observed decrease in alpha diversity was related to a strong negative correlation of *Albugo* with the presence of many bacterial taxa. Stabilization of beta diversity was also reported, i.e., microbiome variability between infected plants was reduced [22]. Moreover, *Saprolegnia*-infected Atlantic salmon (*Salmo salar*) eggs showed significant changes in their microbial landscape, including a decrease in beneficial bacterial taxa like Actinobacteriota, known for their antimicrobial properties. Conversely, there was an increase in opportunistic pathogens, such as *Vibrio*, *Aeromonas*, and *Yersinia*. These changes suggest that *Saprolegnia* infection disrupts the natural balance of the microbiome, making the eggs more susceptible to further infections and reducing overall microbial diversity [2,23]. Furthermore, in vitro and in vivo experiments have shown that specific bacterial isolates originating from the host can play an important role in the host’s protection against pathogens [23,24,25,26,27,28,29]. Bacterial taxa such as *Pseudomonas* and *Aeromonas* have shown inhibitory activity against *S. parasitica* [24,25,30], while isolates of the genus *Frondihabitans* could inhibit *Saprolegnia* attachment to salmon eggs [2]. Selected *Pseudomonas fluorescens* isolates were reported to effectively inhibit *S. parasitica* by stimulating the rainbow trout immune response and producing siderophores and bioactive proteins that suppress the pathogen [30]. The addition of the bacterial strain *Pseudomonas* H6, which is phylogenetically related to *P. fluorescens*, reduced salmon egg mortality caused by *Saprolegnia diclina* in an in vivo study [25].

Trout aquaculture is of global importance due to its contribution to food security, economic growth, environmental sustainability, and rural development [31,32]. In this sector, saprolegniosis is a major and persistent problem, as *S. parasitica* is ubiquitous, and animals are constantly exposed to it [12,20,33,34]. New insights into the interactions between the trout microbiome and *S. parasitica* could contribute to the development of innovative prevention and treatment strategies for saprolegniosis [3,35]. It has been highlighted above that understanding the interactions between certain beneficial microbes and *S. parasitica* can form the basis for developing strategies to protect fish from saprolegniosis through the addition of probiotics to fish feed or water [23,24,30]. A deeper understanding of microbial dysbiosis triggered by the presence of pathogens may also enable us to recognise disease-related shifts in the microbiome at an early stage. Routine monitoring of microbial communities on fish skin and eggs to detect early signs of pathogen presence and microbiome imbalance would enable timely intervention and potentially prevent disease outbreaks or reduce their severity [3,36]. In this context, several *S. parasitica*-specific PCR-based detection assays have recently been developed [33,37,38], while the rapid progress of sequencing technologies allows for easy monitoring of changes in microbial communities in different environments, including aquaculture [39]. Finally, studies focusing on a wide range of hosts, including fish, have shown that the host-associated microbiome can be manipulated using dietary supplements, environmental modifications, or microbial inoculations [36,40]. These interventions could help maintain balanced and healthy microbial communities to aid in the protection of the host from saprolegniosis.

Despite the proven importance of microbial communities in host-pathogen dynamics and the prospects of utilising this knowledge for disease control, previous studies have mostly focused on the direct effects of *S. parasitica* on the host [41,42,43] or on the interactions between axenic cultures of specific bacterial isolates and *S. parasitica* [23,30,44]. In comparison, analyses of the effects of *S. parasitica* on the diversity, composition, and function of microbial communities on fish skin and eggs are scarce [2,23,25]. Our study aims to fill these gaps by applying high-throughput *16S* rRNA gene sequencing to compare the microbial communities on the (i) surface of *Saprolegnia parasitica*-infected vs. healthy trout eggs, and (ii) skin of injured vs. healthy adult trout. This approach has allowed us to identify specific changes in microbial diversity and composition associated with the presence of *S. parasitica*. These new insights into the complex interactions between *S. parasitica* and the trout microbiome could support the development of new strategies for the management of saprolegniosis in aquaculture.

## 2. Materials and Methods

### 2.1. Sample Collection

Adult trout individuals (*N* = 12) were collected at three trout farms in Croatia: Kostanjevac (brown trout, *Salmo trutta*), Radovan (rainbow trout, *Oncorhynchus mykiss*), and Solin (rainbow trout) (Table S1—dataset dataADULTs). Adult trout were captured at the farms and transported to the laboratory in individual containers, where skin swabs were obtained, as described previously [45]. Each individual was classified as either (i) healthy (no external signs of infection or injury) or (ii) injured (animals with external injuries, e.g., lesions). The sampled fish were of market size, typically weighing between 250 and 300 g and measuring between 25 and 30 cm long.

Additionally, at the Kostanjevac and Radovan farms, live, healthy, and fertilised trout eggs were obtained (*N* = 12, Table S2—dataset dataEGGs). Approximately 30 to 50 eggs were separated into subsamples and then placed in sterile Falcon tubes that held 50 mL of farm water each. Following collection, half of the egg samples were infected with *S. parasitica* in a laboratory setting, while the remaining samples served as negative controls. Trout eggs were infected by adding four hemp seeds, overgrown with *S. parasitica* mycelium, to cups containing eggs, while no hemp seeds were added to the negative control samples. In order to prepare the hemp seeds, the mycelial tips of *S. parasitica* were aseptically transferred from glucose-yeast (GY) agar [46] into a Falcon tube containing autoclaved hemp seeds in distilled water and incubated at 18 °C for three days until colonisation with *S. parasitica* hyphae became visible. Swab samples were taken from the egg surface seven days after the infection. After DNA extraction (see Section 2.2.), infection was confirmed with droplet digital PCR (ddPCR) using *S. parasitica*-specific primers (333F and 580R). A comprehensive description of the swab sample procedure, the infection experiment, and the ddPCR reaction is available in our previous manuscript [33].

Aside from the verbal consent provided by the farm managers to gather samples at their farms, no permits were needed for the experimental activities involving eggs and adult trout. Since the experiment was stopped within a week of fertilisation, no approval was required for the experimental *S. parasitica* infection of fertilised trout eggs. Early vertebrate life stages, including fish, are not protected as animals until they can feed themselves, according to EU Directive 2010/63/EU [47]. Independent feeding in trout usually starts eight weeks after fertilisation, which is much after the completion of our trial one week post-fertilisation. As a result, the ARRIVE guidelines and other regulatory frameworks prohibiting animal research do not apply to this infection trial. Regarding the adult trout, no permits were required for sample since the fish were already dead at the time of sampling, as a part of regular harvesting at the fish farms.

### 2.2. DNA Extraction and Determination of S. parasitica Load in the Samples

Using the NucleoSpin^®^ Microbial DNA Kit (Macherey Nagel, Düren, Germany), DNA was extracted from the swabs (i.e., pellets of epibiotic communities from the surface of trout skin and eggs), in accordance with the manufacturer’s protocol, with a few minor changes. Macherey Nagel Bead Tubes Type B were used to lyse the samples using medium strength shaking for 20 min on a Vortex Mixer LSE (Corning, New York, NY, USA). To improve the final sample’s DNA yield and concentration, a second elution was performed from the column using the first 100 μL eluate. Using QuantiFluor ONE dsDNA Dye and agarose gel electrophoresis on a Quantus Fluorometer (Promega, Madison, WI, USA), the quantity and quality of DNA samples were evaluated.

The *Saprolegnia parasitica* load in the samples was determined previously using the developed *S. parasitica*-specific droplet digital PCR (ddPCR) assay [33], and the measurements are reported in Tables S1 and S2.

### 2.3. Microbial Community Analysis by 16S rRNA Gene Sequencing

The composition of the bacterial communities in the DNA recovered from the adult trout and egg swab samples was examined using *16S* rRNA gene amplicon sequencing, which was carried out at Microsynth, Switzerland. The V4 target region (~300 bp) of the *16S* rRNA gene was amplified using *16S* Nextera two-step PCR (including purification and pooling), using forward primer 515F (5′ GTG CCA GCMGCC GCG GTAA 3′) and reverse primer 806R (5′ GGA CTA CHVGGG TWT CTAAT 3′) [48]. Sequencing was performed on an Illumina MiSeq device using the MiSeq Reagent kit v3 (2 × 300 bp paired end reads).

The sequences were analysed using the University of Zagreb University Computing Centre—SRCE advanced computing service and the Quantitative Insights into Microbial Ecology 2 (QIIME2) program [49], 2022.11. Only single-end forward reads were used to identify the bacterial taxa present in the swab samples, since reverse sequences were found to be of inadequate quality and length in some samples during a preliminary examination. Raw sequences were initially demultiplexed and stripped of Illumina adaptor residuals before being loaded into QIIME2 in the sample manifest format. Subsequently, the DADA2 (q2-dada2) plugin was used [50]. DADA2 automatically learns the error model directly from the data by correcting per-base substitution and insertion/deletion errors based on the quality scores assigned to each sequence. With this approach, the pipeline can accurately distinguish between true biological variation and sequencing errors. In addition, the DADA2 pipeline enables the automatic identification and removal of chimeric sequences during the denoising step using a consensus approach. This method compares the sequences within the dataset to detect chimaeras resulting from the combination of two parent sequences during PCR amplification. The chimeric sequences are then labelled and removed. During the DADA2 processing of the dataset, primer sequences were cut out (the first 19 bp), and each sequence was truncated at the point where the median Phred quality score dropped below 28–30 (273 bp), ensuring that only high-quality reads of uniform length were retained for analysis. Then, amplicon sequence variations (ASVs) were assigned and those that occurred in fewer than two samples and at a frequency of less than 0.1% per sample were eliminated from the DADA2-generated feature table through filtering. The taxonomic classification of the ASVs was carried out using the q2 Feature Classifier [51] using the Silva reference taxonomy (v138; [52]). Afterwards, the q2-taxa was used to exclude sequences categorised as mitochondria or chloroplasts from the study. The ASV table was rarefied at 8000 reads in order to include an equal number of reads throughout all samples. All samples from eggs were included, but 15 adult trout samples with <8000 reads were eliminated (Z1, Z3, Z4, Z5, Z6, Z9, Z10, Z11, Z12, Z13, Z14, Z16, B3, B4, RZ11).

### 2.4. Statistical Analysis

Alpha- and beta-diversity indices were used to assess the impact of health status (*S. parasitica*-infected vs. healthy trout eggs and injured vs. healthy adult trout) and sampling location (Kostanjevac, Radovan, or Solin trout farms) on the microbial communities present on the surface of trout eggs or adult trout skin.

Alpha diversity was calculated using the R package *vegan* [53], using three indices: Pielou’s evenness index (measuring how evenly ASVs are represented), the number of observed ASVs (approximation of species richness), and Shannon’s index (combining richness and diversity). For the trout eggs dataset, the effects of health status and location on alpha diversity were analysed using a robust two-factor non-parametric analysis—aligned ranks transformation ANOVA (ART ANOVA, [54])—as traditional parametric ANOVA assumptions were not met. The ART ANOVA model was implemented in R using the *ARTool* library [55], with the following code: *art*(*AlphaDiversity* ~ *Status* + *Location* + *Status:Location*, *data* = *dataEGGs*). For the adult trout dataset, ART ANOVA was not suitable because the requirement for the F values of ANOVAs on aligned responses to be zero was not met. Instead, the non-parametric two-factor Scheirer–Ray–Hare test was used [56]. The R implementation in the *rcompanion* library [57] was applied, with the following code: *scheirerRayHare*(*AlphaDiversity* ~ Status*Location, *data* = *dataADULTs*).

Three phylogenetic measures of beta diversity were used: (i) weighted UniFrac distance (a metric determined by comparing the relative abundance of different taxa), (ii) unweighted UniFrac distance (a qualitative assessment based on the presence or absence of different taxa) [58,59], and (iii) Bray–Curtis distance (a measure of compositional dissimilarity between samples, based on the counts of shared taxa) [60,61]. Beta-diversity indices using UniFrac distances were calculated with QIIME2 [49] and the R package *phyloseq* (Version 1.46.0) [62]. Indices using Bray–Curtis distance were calculated using the *vegan* package [53]. The effects of health status and sampling location on beta diversity were analysed using permutational analysis of variance (PERMANOVA) in the vegan package, with the following code: *adonis2*(*BetaDiversity* ~ *Status* + *Location*, *permutations* = *9999*).

The influence of health status and location on the presence of *S. parasitica* on the surface of eggs and the skin of adult individuals was assessed using the ART ANOVA model and the Scheirer–Ray–Hare test, respectively. Finally, the analysis of the most prevalent genera and phyla, as well as all data transformations and visualizations, was conducted using the R package *phyloseq* [62].

## 3. Results

High-throughput sequencing of the V4 region of the *16S* rRNA genes from the swab samples yielded a total of 1,750,831 reads. After processing with DADA2 and filtering the resulting feature table, 921,936 reads from 24 samples (12 from eggs and 12 from adult trout) were retained. This analysis identified a total of 176 ASVs from eggs and 251 from adult trout.

### 3.1. Bacterial Communities on the Surface of Trout Eggs Infected with S. parasitica Compared to Healthy Eggs

Trout eggs collected from the Kostanjevac and Radovan fish farms, belonging to brown and rainbow trout, respectively, were divided into two groups. One group was infected with the pathogen *S. parasitica* in the laboratory, while the other was used as a non-infected negative control. The health status of the eggs was verified by comparing the abundance of *S. parasitica* in the infected and non-infected egg swabs, and it was confirmed that the infected eggs had a significantly higher *S. parasitica* load than that of the non-infected controls (*p* < 0.01) (Table S3). The infected and non-infected groups were then further compared to analyse the differences in their microbial communities in detail.

**Figure 1:**
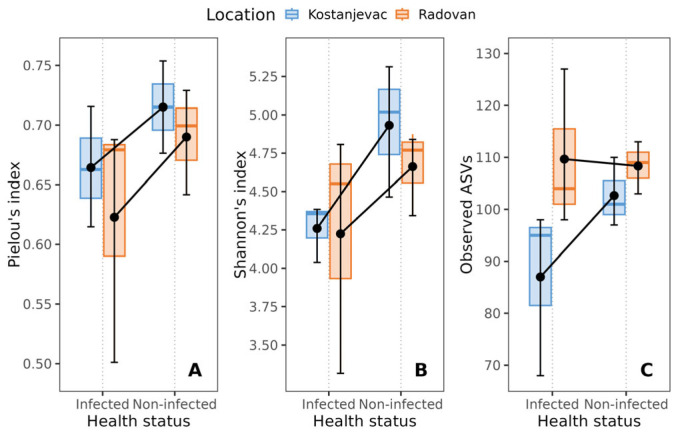
Effect of the health status of trout eggs (*S. parasitica*-infected and non-infected) on alpha diversity indices: (**A**) Pielou’s index, (**B**) Shannon’s index, (**C**) observed ASV’s. The black error bars indicate the mean value, with the corresponding 95% confidence intervals.

Overall relatively low values of alpha diversity indices were found: the average Pielou’s evenness index was 0.68, the average Shannon’s index was 4.52, and the average observed ASVs was 102. However, non-infected trout eggs exhibited higher bacterial diversity, as evidenced by higher Pielou’s evenness and Shannon’s indices, compared to that observed for eggs infected with *S. parasitica* in the laboratory (Figure 1). Although neither of the *p*-values (*p* ≈ 0.07, Table S4) reached conventional statistical significance (*p* < 0.05), they suggest a tendency towards higher diversity in the non-infected group, consistent with substantial effect sizes of health status (η_S_^2^ = 0.346 and η_P_^2^ = 0.350, Table S4). While location was not significantly associated with Pielou’s evenness and Shannon’s indices (Table S4, *p* > 0.05), it was significantly associated with bacterial diversity, as measured by the number of observed ASVs (*p* < 0.05, Table S4).

**Figure 2:**
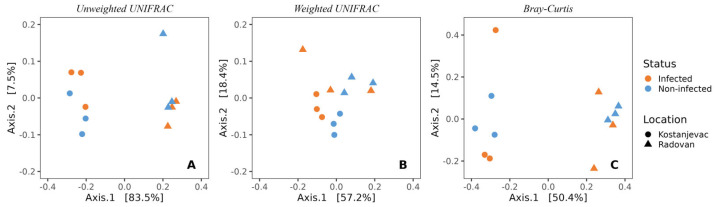
Beta-diversity of the bacterial communities present on the surface of trout eggs, depending on the infection status (*S. parasitica*-infected or healthy) and sampling location (Kostanjevac or Radovan trout farm): (**A**) unweighted UNIFRAC; (**B**) weighted UNIFRAC; (**C**) Bray–Curtis.

Beta diversity analysis revealed that the sampling locations (Kostanjevac and Radovan farms) were significantly associated with the composition of microbial communities on the egg surface. This was evident both from weighted (*p* < 0.05) and unweighted UniFrac (*p* < 0.001), as well as from the Bray–Curtis analysis (*p* < 0.001) (Figure 2, Table S5). However, the weighted UniFrac analysis indicated that the influence of *S. parasitica*-infection status on microbial community composition approached statistical significance (*p* = 0.10, Table S5), suggesting a potential link between the abundance of specific bacterial taxa and the presence or absence of *S. parasitica*.

**Figure 3:**
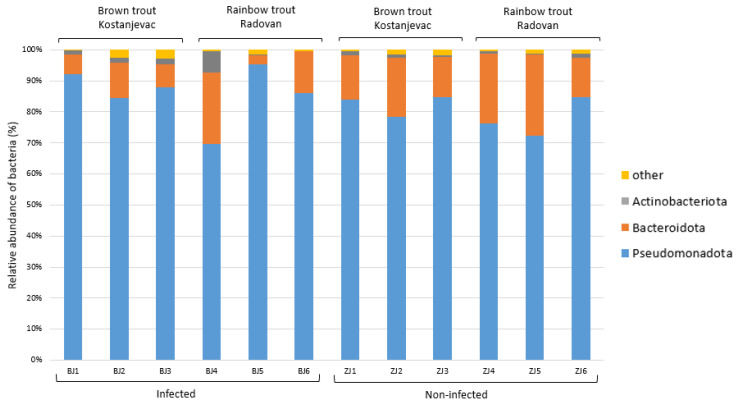
Relative abundance (%) of the bacterial phyla in the egg samples (*N* = 12). Bacterial phyla with a relative abundance >5% in at least one sample are shown, while the others were combined and labelled as “other”.

**Figure 4:**
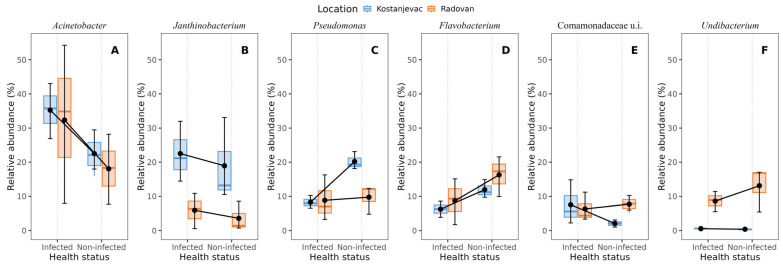
Bacterial genera with >5% average relative abundance on the egg surface in relation to the infection status (*S. parasitica*-infected or non-infected) and location (Kostanjevac or Radovan fish farm): (**A**) *Acinetobacter*, (**B**) *Janthinobacterium*, (**C**) *Pseudomonas*, (**D**) *Flavobacterium*, (**E**) unidentified genus from the family Commamonadaceae, (**F**) *Undibacterium*. The black error bars indicate the mean value, with the corresponding 95% confidence intervals.

Taxonomic analysis of the ASVs in the collected samples revealed that the most abundant bacterial phyla in the egg samples were Pseudomonadota and Bacteroidota, with an average abundance of 83% and 14%, respectively (Figure 3). We have further examined whether the bacterial genera present with a >5.0% average relative abundance in the egg samples (i.e., *Acinetobacter*, *Janthinobacterium*, *Pseudomonas*, *Flavobacterium*, unidentified genus from the family Commamonadaceae, and *Undibacterium*) were present in significantly different abundances on the surface of infected and non-infected eggs (Table S6, Figure S1). The analysis indicated that *Acinetobacter* and *Janthinobacterium* were more prevalent in the infected group (Figure 4A,B), although this difference was not statistically significant (*p* > 0.05), while *Pseudomonas* and *Flavobacterium* were more abundant in the non-infected group (Figure 4C,D), with statistically significant differences confirmed for both of these genera (Table S6, *p* < 0.05). For *Pseudomonas*, a significant interaction was also observed between health status and location (*p* < 0.05), indicating that the effect of infection on *Pseudomonas* abundance varied between the Kostanjevac and Radovan fish farms. Furthermore, location was significantly associated with the abundance of *Janthinobacterium* (*p* < 0.001) and *Undibacterium* (*p* < 0.001).

### 3.2. Bacterial Communities on the Trout Skin in Relation to S. parasitica Load

Adult rainbow and brown trout, both healthy and injured, were collected from the Kostanjevac, Radovan, and Solin fish farms. Injured trout individuals had a higher *S. parasitica* load than that of the healthy specimens, although this difference approached, but did not reach, statistical significance (*p* = 0.07, Table S7). Here, our aim was to analyse the differences in microbial communities present on the skin of healthy and injured adult trout.

As for the egg samples, the alpha diversity of bacterial communities on the skin of adult trout was low, as indicated by the average values of the Pielou’s evenness index (0.55), the average Shannon’s index (3.46), and the average observed ASVs (79). There was no discernible trend in alpha diversity indices between groups based on sampling location or health status (Table S8, *p* > 0.05), in contrast to the results for the egg samples, in which the diversity of microbial communities was lower in the *S. parasitica*-infected eggs.

**Figure 5:**
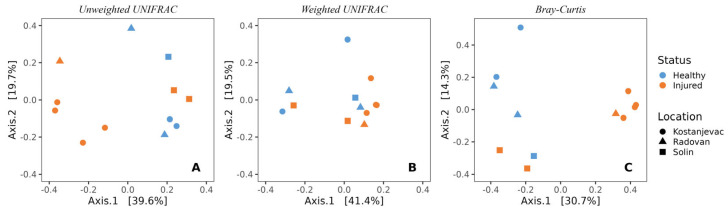
Beta-diversity of the bacterial communities present on the skin surface of adult trout individuals, depending on the health status (injured or healthy) and location (Kostanjevac, Radovan, or Solin fish farm): (**A**) unweighted UNIFRAC; (**B**) weighted UNIFRAC; (**C**) Bray–Curtis.

In line with the beta diversity findings for the egg samples, the sampling location was also significantly associated with the composition of microbial communities on the surface of the adult trout (Figure 5, Table S9, unweighted UniFrac: *p* < 0.01; Bray–Curtis: *p* < 0.05). Health status was also significantly associated with the microbial communities of adult trout (unweighted UniFrac: *p* < 0.01; Bray–Curtis: *p* < 0.01, Table S9), indicating the presence of specific bacterial species associated with the injured adult trout, along with *S. parasitica* (Figure 5).

**Figure 6:**
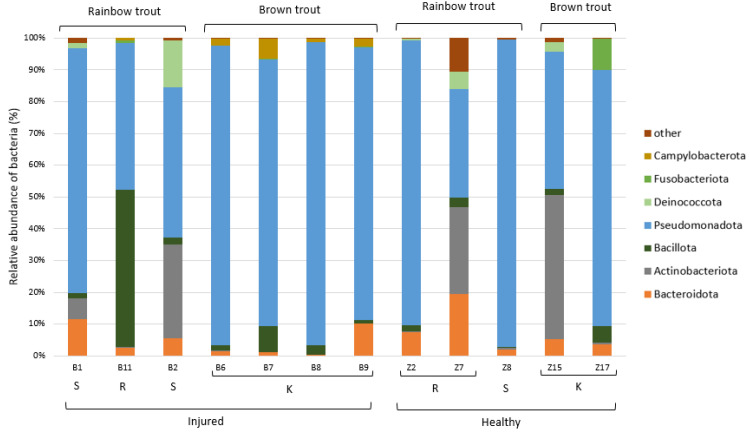
Relative abundance (%) of the bacterial phyla in the adult trout samples (*N* = 12). Bacterial phyla with relative abundance >5% in at least one sample are shown, while the others were combined and labelled as “other”. K—Kostanjevac, R—Radovan, and S—Solin.

The taxonomic analysis of the ASVs in the collected samples indicated that the most dominant phylum present in the skin microbiome of the adult trout was Pseudomonadota (75%), followed by the less-represented Bacteroidota (10%), Actinobacteriota (5%), and Bacillota (3%) (Figure 6). Dominant bacterial genera present with >5.0% average relative abundance were *Aeromonas*, *Pseudomonas*, *Acinetobacter*, and an unidentified genus from the family Yersiniaceae. We have evaluated whether these most abundant bacterial genera were present in significantly different abundances in relation to the health status of the adult trout, and only genus *Aeromonas* showed significantly higher abundance on the skin of the injured trout (*p* < 0.01, Table S10, Figure S2).

## 4. Discussion

Our study is the first to utilise high-throughput *16S* rRNA gene sequencing to analyse the bacterial communities on the surface of trout eggs and the skin of adult trout in relation to the presence of *S. parasitica*. The obtained results show that saprolegniosis is associated with shifts in host-associated bacterial communities, including changes in abundance of some bacterial taxa, as well as overall microbiome diversity and composition. Further, bacterial communities were also associated with the location of sampling, highlighting the substantial effect of the environment on the composition of trout surface microbiomes.

*Saprolegnia parasitica* is an oomycete pathogen whose life cycle comprises a sexual and an asexual part, the latter being responsible for the infection of the host [13,15,21]. In the asexual cycle, the sporangia produce free-swimming biflagellate zoospores that allow the pathogen to spread to new environments and to be attracted to the host by chemotaxis. After reaching the surface of the host, they encyst, and the cysts begin to germinate to invade the host organism, where the mycelium develops and causes the disease symptoms. The host immune response serves as a defence against invasion by the pathogen, including the activation of innate immune defences such as proinflammatory cytokines and antimicrobial peptides [63,64,65,66]. In addition to host immunity, microbial communities that form biofilms on the surface of trout skin and eggs can help defend the host against pathogens, as they represent an initial barrier to pathogen invasion [67,68,69]. For example, it has been shown that certain bacterial strains originating from the skin/egg surface of the host can inhibit *S. parasitica* [2,23,24,25,30]. However, if the defence systems are overridden by the pathogen, this leads to the development of the disease, which is often accompanied by microbial dysbiosis [5,68,69,70]. This is well documented in mammalian systems [71], but the effects of *S. parasitica* on the diversity, composition, and function of microbial communities on fish skin and eggs have scarcely been studied [2]. 

In our study, non-infected trout eggs exhibited higher bacterial diversity compared to those with saprolegniosis, and although the difference was not statistically significant, a trend was clearly visible. Similarly, Liu et al. [2] reported a higher richness of microbial communities on the surface of healthy salmon eggs compared to those infected with saprolegniosis. This suggests that the disease susceptibility of salmonid eggs is defined, to some extent, by their microbiome, in addition to other factors like genetics, developmental stage, and the environment, especially when keeping in mind that the immune system of the eggs is immature and thus, dependent, to a large extent, on other defences [72]. In comparison, in our study, the skin microbiome of the adult trout did not show a similar trend of a decrease in richness related to *S. parasitica* presence. This might be explained by the fact that the epidermis and mucus of adult fish, compared to those of the egg surface, are immunologically more active barriers, equipped to fight the colonization by *S. parasitica* and other pathogens [73]. However, changes in the alpha diversity indices of fish-skin microbiomes due to infection by bacterial and other pathogens have been previously described, mainly (but not exclusively) reporting a decrease in microbial diversity [74,75,76]. It is therefore possible that if the *S. parasitica* load, host immune suppression, and/or sample size were higher in our dataset, we would have observed a statistically significant association of *S. parasitica* presence with alpha diversity. However, further research is needed to confirm this hypothesis.

Further, analyses of beta diversity showed a significant association of both sampling location (different fish farms) and health status (presence or absence of *S. parasitica*) on the composition of microbial communities on the surface of eggs and adult trout. This highlights the substantial correlation of abiotic and biotic environment with the composition of host-associated microbiomes and is consistent with the results of previous studies [77,78]. For instance, McMurtrie et al. [78] reported significant differences in microbial communities on the skin of tilapia originating from different aquaculture ponds. They demonstrated that only slightly more than 1% of pond water prokaryotic ASVs were found across all seven pond sites, indicating limited species dispersal and/or unique micro-ecologies between the ponds. Similarly, Sylvain et al. [79] showed that the skin mucus microbiome of three ecologically and phylogenetically contrasting teleost species is predominately defined by environmental factors, such as physicochemistry and bacterioplankton community structure, in contrast to gut microbial communities, which are more species-specific. Therefore, although we did not analyse environmental microbiomes in the sampling locations, we can assume that different fish farms exhibit distinct environmental conditions, such as differences in feeding regimes and the physical and chemical parameters of the water, eventually leading to differences in the egg/skin microbiomes. In addition, brown trout were reared in the Kostanjevac fish farm and rainbow trout in the Radovan and Solin farms, so species-specific factors could also have played a role in the variability of the microbiomes. Finally, the immune response to the presence of *S. parasitica* was probably different among various fish individuals, developmental stages, and species. These differences probably helped to shape the microbiomes of the trout analysed in our study [80,81], although the immune response was not analysed.

Further, in agreement with our results, the presence of pathogens was often shown to be associated with the fish microbiome structure, and it was noted that the fish skin and the egg surface are more prone to dysbiosis than are the internal organs, such as the gut [70]. Recognizing the signatures of dysbiosis in different fish species and organs associated with the presence of different pathogens is crucial for developing effective disease-control strategies. However, only a few previous studies have analysed the effects of *Saprolegnia* on the salmonid skin and egg microbiome [2,25]. In our study, *Pseudomonas* and *Flavobacterium* were significantly more abundant in the non-infected egg samples, while *Aeromonas* was significantly more abundant on the skin of the injured trout. All three of the listed genera include species pathogenic to salmonids, like *Pseudomonas anguliseptica* [37], *Flavobacterium psychrophilum* [82], and *Aeromonas salmonicida* [83]. On the other hand, members of these genera were often isolated from healthy salmonid skin or egg surfaces and were shown to inhibit oomycetes. For instance, *Flavobacterium johnsoniae* strain GSE09 can form biofilms and produce various bioactive compounds that inhibit the pathogenic oomycete *Phytophthora capsica* [84]. *Pseudomonas* spp., mostly from the *fluorescens* group, were repeatedly proven to be potent inhibitors of *S. parasitica* and other aquatic oomycetes [25,29,30,44,85]. Carbajal et al. [23,44] demonstrated that isolates from the genera *Aeromonas* and *Pseudomonas* exhibited strong inhibitory activity against *S. parasitica*, were non-pathogenic to rainbow trout, and could reduce the adhesion of *S. parasitica* zoospores and cysts to skin mucus. Therefore, we propose that different bacterial species from these bacterial genera present on trout skin/eggs are involved in complex and variable interactions with both the host and other microbes. On the one hand, many bacteria, including *Pseudomonas*, *Aeromonas*, and *Flavobacterium* spp., are opportunistic pathogens that can switch from a commensal to a pathogenic lifestyle. On the other hand, they may be involved in inter-microbial antagonism and exhibit defence mechanisms to outcompete or inhibit other potentially pathogenic microbes, such as *S. parasitica*, that attempt to invade the same host.

## 5. Conclusions and Future Directions

We have shown that the presence of *S. parasitica* is associated with the changes in the diversity and structure of the trout skin/egg microbiome, as well as with altered abundances of some microbial genera, such as *Pseudomonas* and *Flavobacterium*. In the future, our results should be further tested using larger sample sizes, since the small number of analysed samples presents a major limitation of the current study. The effects of other parameters known to influence the composition of the microbiome, such as physicochemical factors of the environment (e.g., water temperature, light, and chemistry) and the parameters of the individual fish (such as immune response, including humoral and cellular immune response parameters; sex; age; fish and egg size; etc.), should also be considered. Finally, complex tripartite interactions between (i) bacterial isolates from a selected genus (that could range from obligatory pathogens, to opportunistic pathogens, to host-beneficial mutualists), (ii) *S. parasitica* pathogens, and (iii) selected fish hosts, should be analysed. The mechanisms explaining the interactions of these bacteria with *S. parasitica* could be deciphered in vitro using microbiological dual culture assays; molecular analyses, such as genome mining, transcriptomics, and proteomics; as well as metabolomic profiling to identify putative bioactive metabolites of bacterial origin. In addition, the immune response of trout in the presence of *S. parasitica*, with or without the selected bacteria, should be analysed in vivo. Such experiments would further unravel the complexity of the saprolegniosis ecology, relevant in both wild environments and in aquaculture, opening the possibilities for the development of sustainable control of this disease.

## Data Availability

Raw *16S* rRNA sequence data generated and analysed within this study are freely available at the National Centre for Biotechnology Information (NCBI), under the BioProject accession number PRJNA1138216.

## Supplementary Materials

The following supporting information can be downloaded at: https://www.mdpi.com/article/10.3390/microorganisms12081733/s1, Figure S1: Relative abundance (%) of the bacterial families in the collected egg samples (*N* = 12). Bacterial families with relative abundance >5% in at least one sample are shown, while the remaining were pooled and indicated as “other”. K—Kostanjevac, R—Radovan, and S—Solin; Figure S2: Relative abundance (%) of the bacterial families in the collected adult trout samples (*N* = 12). Bacterial families with relative abundance >5% in at least one sample are shown, while the remaining were pooled and indicated as “other”. K—Kostanjevac, R—Radovan, and S—Solin; Table S1: Sample dataset for adult trout; ND—not determined; Table S2: Sample dataset for eggs; ND—not determined; Table S3: Non-parametric analysis of *Saprolegnia* load on the egg surface in relation to infection status (infected or non-infected) and location (Kostanjevac or Radovan fish farm) using aligned ranks transformation ANOVA. Partial eta squared (η^2^) is specified as the measure of effect size. *Saprolegnia* abundance was log-transformed (1 + log(*Saprolegnia*)) prior to analysis; Table S4: Non-parametric analysis of alpha-diversity indices of microbial communities on the egg surface in relation to infection status (*S. parasitica*-infected or non-infected) and location (Kostanjevac or Radovan fish farm) using aligned ranks transformation ANOVA. Partial eta squared (η^2^) is specified as the measure of effect size; Table S5: Permutational analysis of beta-diversity of ASVs on egg surface in relation to infection status (*S. parasitica*-infected or non-infected) and location (Kostanjevac or Radovan fish farm) using three distance metrics; Table S6: Non-parametric analysis of most abundant bacterial genera (>5.0% average relative abundance) on the egg surface in relation to infection status (*S. parasitica*-infected or non-infected) and location (Kostanjevac or Radovan fish farm) using aligned ranks transformation ANOVA. Partial eta squared (η^2^) is specified as the measure of effect size; Table S7: Non-parametric analysis of *Saprolegnia* load on the trout skin in relation to health status (injured or healthy) and location (Kostanjevac, Radovan, or Solin fish farm) using Scheirer–Ray–Hare test; Table S8: Non-parametric analysis of alpha-diversity indices of microbial communities on the trout skin in relation to health status (injured or healthy) and location (Kostanjevac, Radovan, or Solin fish farm) using Scheirer–Ray–Hare test; Table S9: Permutational analysis of beta-diversity of ASVs on trout skin in relation to health status (injured or healthy) and location (Kostanjevac, Radovan, or Solin fish farm) using three distance metrics; Table S10: Non-parametric analysis of most abundant bacterial genera (>5.0% average relative abundance) on the trout skin in relation to health status (injured or healthy) and location (Kostanjevac, Radovan, or Solin fish farm) using Scheirer–Ray–Hare test.
