# Supplementary material for: Characterization of Bacterial Communities on Trout Skin and Eggs in Relation to Saprolegnia parasitica Infection Status
Source: Microorganisms. 2024 Aug 22;12(8):1733. doi: 10.3390/microorganisms12081733 (PMC11357440; doi:10.3390/microorganisms12081733)
Supplement: Supplementary file 1 [file microorganisms-12-01733-s001.zip › Pavic et al_R1_Supplementary Figures.pdf]

Supplementary material of the manuscript entitled 'Characterization of bacterial communities on trout skin and eggs in relation to *Saprolegnia parasitica* infection status' by Dora Pavić, Sunčana Geček, Anđela Miljanović, Dorotea Grbin and Ana Bielen

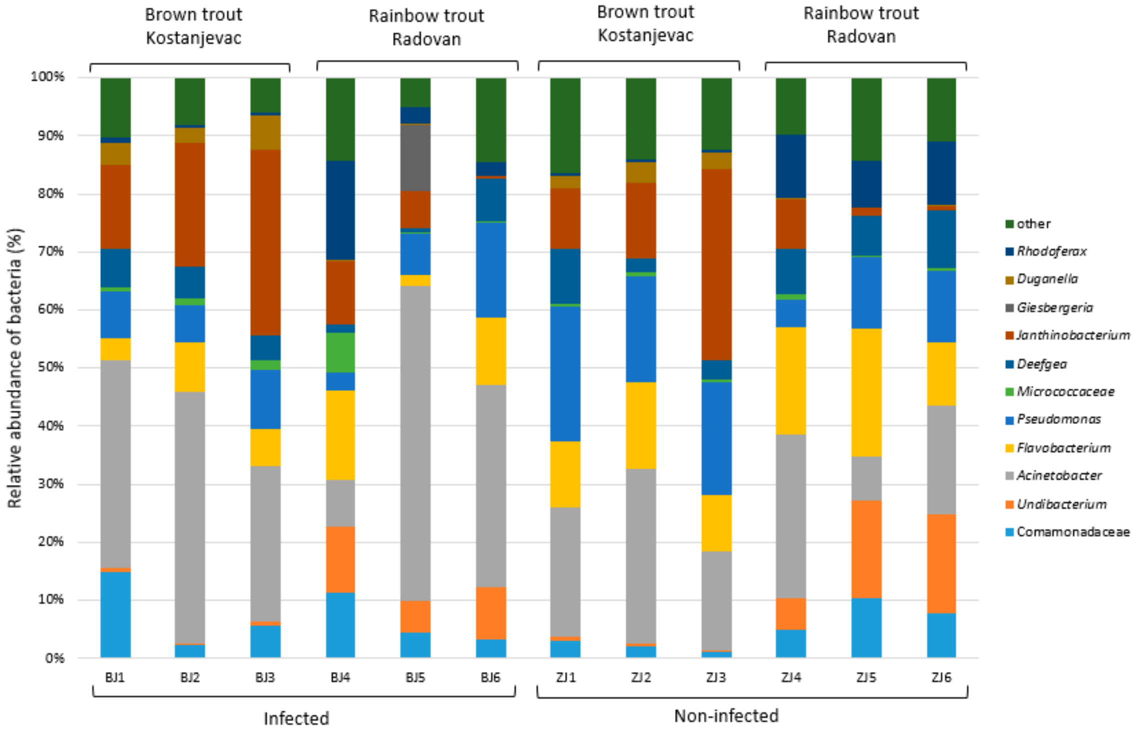

Figure S1. Relative abundance (%) of the bacterial families in the collected egg samples (N=12). Bacterial families with relative abundance > 5% are shown, while the remaining were pooled and indicated as “other”. K – Kostanjevac, R – Radovan and S – Solin.

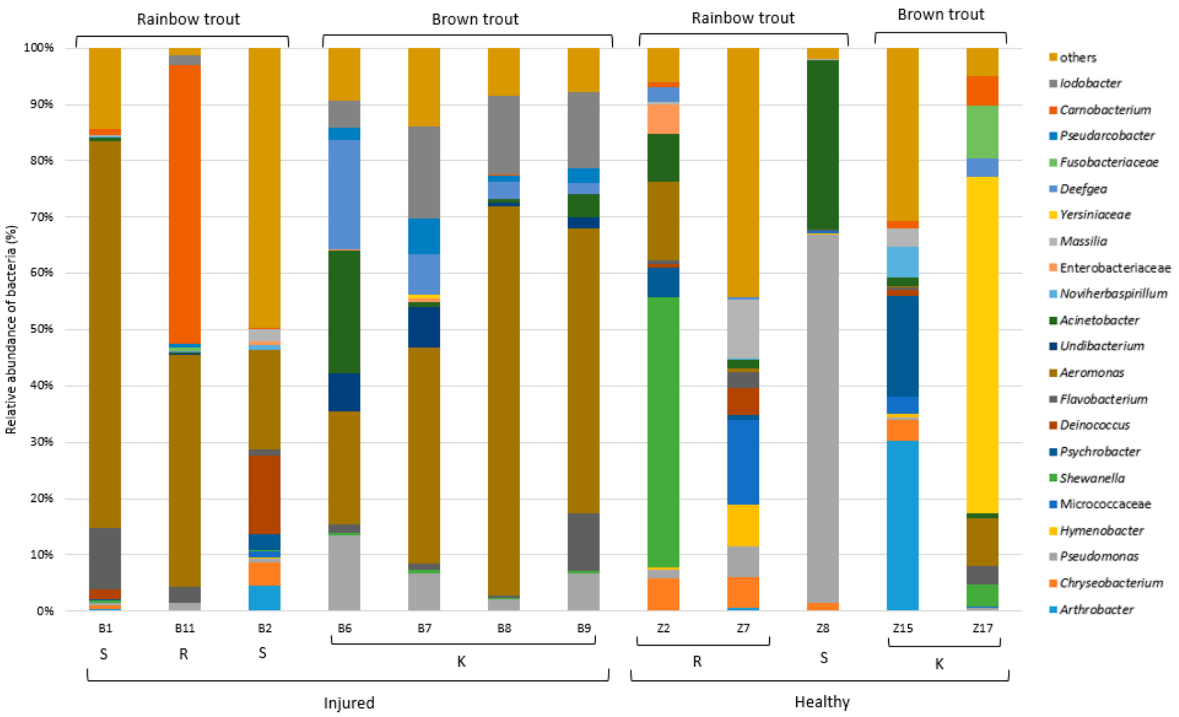

Figure S2. Relative abundance (%) of the bacterial families in the collected adult trout samples (N=12). Bacterial families with relative abundance > 5% are shown, while the remaining were pooled and indicated as "other". K – Kostanjevac, R – Radovan and S – Solin.
